# CORA: An Open-Source Software Tool for Combinational Regularity Analysis

**DOI:** 10.1177/08944393241275640

**Published:** 2024-08-28

**Authors:** Lusine Mkrtchyan, Alrik Thiem, Zuzana Sebechlebská

**Affiliations:** 1 30731 University of Lucerne, Switzerland

**Keywords:** Boolean algebra, Coincidence Analysis, Combinational Regularity Analysis, Configurational Comparative Methods, Google Colaboratory, Python, Qualitative Comparative Analysis

## Abstract

Modern Configurational Comparative Methods (CCMs), such as Qualitative Comparative Analysis (QCA) and Coincidence Analysis (CNA), have gained in popularity among social scientists over the last thirty years. A new CCM called Combinational Regularity Analysis (CORA) has recently joined this family of methods. In this article, we provide a software tutorial for the open-source package **CORA** , which implements the eponymous method. In particular, we demonstrate how to use **CORA** to discover shared causes of complex effects and how to interpret corresponding solutions correctly, how to mine configurational data to identify minimum-size tuples of solution-generating inputs, and how to visualize solutions by means of logic diagrams.

## Introduction

Configurational Comparative Methods (CCMs) constitute a family of empirical research methods for causal inference that have gained in popularity among social scientists over the last thirty years (Rihoux & Ragin, 2009; Thiem, 2022b). While the inferential foundations of modern CCMs lie within the class of regularity theories of causation (Psillos, 2009), and in particular the INUS Theory (Mackie, 1965, 1980), their mathematical operations are firmly anchored in Boolean algorithms. CCMs can thus be distinguished most clearly from other formalized methods of data analysis by their reliance on the laws of Boolean and Post algebra (Mkrtchyan et al., 2023; Thiem et al., 2016).

For many years, the two most advanced CCMs have been Qualitative Comparative Analysis (QCA; Ragin, 1987, 2000, 2008) and Coincidence Analysis (CNA; Baumgartner, 2009; Baumgartner & Ambühl, 2020). QCA-based studies have long been published in numerous disciplines, mainly business, management and organization studies, political science and sociology (Rihoux et al., 2013; Thiem, 2022b; Wagemann et al., 2016). Although CNA is appreciably more powerful than QCA in its analytical capabilities (Baumgartner & Ambühl, 2020), applications have only begun to appear towards the late 2010s, and almost exclusively in health-related research so far (Whitaker et al., 2020).

A third method called Combinational Regularity Analysis (CORA) has recently joined the fold (Mkrtchyan et al., 2023; Sebechlebská et al., 2023; Thiem et al., 2022, 2024). Most importantly with regard to configurational data analysis, CORA has introduced the possibility to analyze multiple outcomes as well as the conjunctions of these outcomes simultaneously. To this end, CORA generalizes the process of Boolean optimization from the level of single outcomes to entire systems of outcomes. In this way, not only can the causes of individual effects be found, but also the causes of all possible combinations of these effects. For instance, the concept of multi-morbidity, which has assumed increased importance in medical, health and psychological research over the last decade (e.g., King et al., 2018; Suls & Green, 2019), describes the simultaneous presence of multiple diseases in a patient, such as diabetes and depression. A risk factor or combination of risk factors may impact on only one disease at a time, but not the other, or the conjunction of both diseases. In such contexts, CORA represents the only CCM that is able to correctly infer such relations from corresponding data.

A second difference of CORA to QCA and CNA is its in-built option to address the issue of model ambiguity by a data-mining procedure that progresses through tuple-selections of input factors. The idea behind this approach is that any explanatory model that is found with a given number of inputs will, *ceteris paribus*, also always be found in an analysis with only those inputs involved in this model. More precisely, if CORA cannot identify a fitting model with some set of combinations of inputs, it adds one more input to this set. This approach represents a configurational version of Occam’s Razor, which holds that explanations involving fewer variables are, *ceteris paribus*, to be preferred over explanations that are more complex (Feldman, 2016).^1^

Third, CORA is the only configurational method to date that offers a consistent and effective way of visualizing its solutions. To do so, it draws on logic diagrams (Thiem et al., 2023). Originally, logic diagrams were exclusively used in electrical engineering for representing Boolean functions in general, and switching circuits in particular, but since the early 2000s, they have also become widespread in some subfields of biology (Wang & Buck, 2012). In fact, some prominent researchers in causal inference and artificial intelligence even hold that logic diagrams ”capture …the very essence of causation” (Pearl, 2009, p. 415).

In this article, we provide a software tutorial for the open-source Python package **CORA** , which implements the eponymous method. First, we give a general overview of the software landscape for configurational data analysis, and outline the features that make **CORA** distinct. Subsequently, we provide a step-by-step guide on the use of **CORA** , with a focus on the three core features outlined above. Specifically, we demonstrate how to use **CORA** to process configurational data that feature more than one endogenous factor, how to mine such data to identify the smallest sets of exogenous factors that generate a solution, and how to visualize solutions by means of logic diagrams. In the remainder of this article, we use a type-writer font when referring to software (e.g., **CORA** ), and a regular font when referring more generally to a method (e.g., CORA). Exogenous factors we also call more succinctly ”inputs”, and endogenous factors ”outputs”. A level or a conjunction of levels of an exogenous factor is also referred to as a ”condition”, a level or a conjunction of levels of an endogenous factor as an ”outcome”.

## Software Overview

The software landscape for CCMs has grown considerably in recent years. How **CORA** compares to other software packages currently available for configurational data analysis is shown in Table 1. For QCA, CNA and CORA, applied researchers can draw on at least one software package ( **CORA** : Sebechlebská et al., 2023; **cna** : Ambuehl & Baumgartner, 2022; **QCApro** : Thiem, 2018; **QCA** : Duşa, 2022; **fs/QCA** : Ragin & Davey, 2019; **Tosmana** : Cronqvist, 2019). For QCA in particular, there currently exist several packages. A range of products to choose from is usually to the benefit of consumers, but the problem with the multitude of QCA software is that procedures are not harmonized, in consequence of which it is not guaranteed that users will obtain the same results with two different packages even though they draw on the same data and set the same analytical parameters. This, in turn, impedes scientific progress and replicability (Baumgartner & Thiem, 2017; Thiem & Duşa, 2013a; Thiem et al., 2020). The reason for this situation is the general lack of agreement in the QCA community about what the actual search target of QCA is and how QCA should go about realizing its competing search targets (Baumgartner, 2022; Baumgartner & Thiem, 2017, 2020; Thiem & Mkrtchyan, 2024b). In contrast, CORA and CNA have clearly defined search targets, as a result of which their respective software packages **CORA** and **cna** produce solutions that are guaranteed to be coherent.

**Table 1.** Overview of Current Software Packages for configurational Data Analysis.

Tool	**CORA**	**cna**	**QCApro**	**QCA**	**fs/QCA**	**Tosmana**
Method	CORA	CNA	QCA	QCA	QCA	QCA
Current version	2.0.11	3.4.0	1.1.2	3.17	3.1b	1.61
Environment	Python	R	R	R	stand-alone	stand-alone
	Colab Notebook					
Open source	yes	yes	yes	yes	no	no
License	GPL-3	GPL-3	GPL-3	GPL-3		
Language	Python	R	R	R	C++	C#
	C++	C++	C++	C++		.NET
User interface	command-line,	command-line	command-line	command-line	graphical	graphical
	graphical			graphical		
Data formats	csv, xlsx	R formats	R formats	R formats	csv, dat,	csv, dat,
					raw, txt	xml
Factor types	bivalent	bivalent	bivalent	bivalent	bivalent	bivalent
	multivalent	fuzzy	fuzzy	fuzzy	fuzzy	fuzzy (limited)
		multivalent	multivalent	multivalent		multivalent
Algorithms	ON-DC	cna	eQMC	eQMC	QMC	graph-based agent
	ON-OFF			CCubes		
Fit statistics	inclusion	consistency	inclusion	consistency	consistency	consistency
	raw coverage	raw coverage	raw coverage	raw coverage	raw coverage	raw coverage
	unique coverage	unique coverage	unique coverage	unique coverage	unique coverage	unique coverage
Graphics	logic diagrams	R graphics	R graphics	Venn diagrams,	bivariate plots	Tosmana maps
				bivariate plots		bivariate plots

In the family of software packages for configurational data analysis, **CORA** is the only package almost completely written in Python. It can be used either in a purely code-based Python environment or in a graphical Google Colab environment. In contrast, what **CORA** has in common with **cna** or **QCApro** , for example, is the fact that all its code is open source under a GPL-3 licence. As part of a general drive towards more open scientific practices, open source software is an integral element whose importance cannot be stressed enough.^2^ As the idea of open science has been integral to CORA’s development from the start, all components of **CORA** are hosted on GitHub - a widely-used web-based service for the version control of software development projects (Blischak et al., 2016; Perkel, 2016).

With regard to data formats, **CORA** accepts csv (comma-separated value) and xlsx (Microsoft Excel) files. Especially csv files can be easily imported into all software packages for configurational data analysis. More notably, however, configurational data suitable for CORA include only bi- or multivalent factors. Due to the multitude of analytical and conceptual problems associated with the use of fuzzy sets (e.g., Cooper & Glaesser, 2011; Thiem & Duşa, 2013b, p. 64), CORA does not offer functionality for this type of data. What is more, however, multivalent factors allow for a level of nuances in configurational inferences that the use of bivalent or fuzzy sets does not permit. Irrespective of whether users draw on crisp-set QCA or fuzzy-set QCA, crisp-set CNA or fuzzy-set CNA, respectively, the type of inferences in their solutions will necessarily remain binary.^3^ CORA, in contrast, has been explicitly designed to allow for multivalent factors on both the input and the output side, even with multiple outputs, thereby making it possible to generate fine-grained inferences that are only limited by the number of factor levels and computational constraints.

Algorithms play an important role in configurational data analysis, as they determine the output and the speed with which that output will be obtained. In CORA, users currently have the choice between two different optimization algorithms, one that optimizes on the basis of positive terms and don’t care terms (ON-DC)—and another that optimizes based on positive and negative terms (ON-OFF). Algorithms of the latter type enjoy significant computational advantages when the set of don’t care-terms is large relative to the set of off-terms. As these two algorithms are equivalent, they will always produce exactly the same solutions.^4^

All fit statistics in CORA are conceptually equal to those in QCA or CNA (inclusion and consistency are conceptually identical). However, when dealing with multiple outputs simultaneously, some generalizations are needed that we will explain in more detail further down.

The last difference between CORA and QCA or CNA is its incorporation of logic diagrams. Due to the limited set of logic design symbols required to visualize CORA’s solution and their purpose-tailored layout, logic diagrams are called ”logigrams” in CORA. For producing logigrams, **CORA** builds on a sister package named **LOGIGRAM** (Sebechlebská et al., 2023). The **LOGIGRAM** module is an integral part of **CORA** , but it can also be used as a stand-alone application for the general visualization of two-level Boolean functions. Therefore, **LOGIGRAM** also has its own GitHub page.^5^ The clear advantage of logic diagrams, whether in the CORA-specific form of logigrams or else, over other means of visualization used in configurational data analysis, such as Venn diagrams, is that they easily convey the make-up of INUS structures. Venn diagrams, in contrast, allow one to easily see which types of cases are compatible with a certain Boolean function, but they do not convey the structure of the function itself. What represents a cause, what an effect, and the way in which cause(s) and effect are related, cannot be read from Venn diagrams (Thiem et al., 2023, 2024).

## General Description of **CORA**

**Figure 1.**
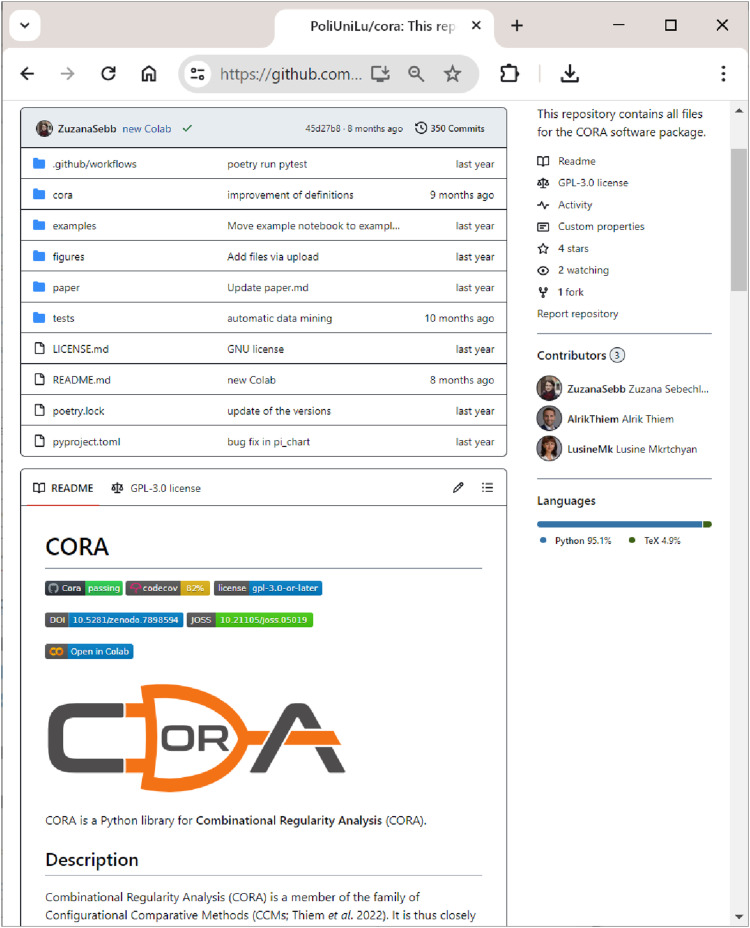
**CORA** ’s GitHub page.

All components of **CORA** are hosted on GitHub at https://github.com/PoliUniLu/cora. GitHub is a widely-used web-based service for the version control of software development projects. Inspection of code, user feedback, bug reports etc. are all handled via this page. Figure 1 shows a snapshot of the upper part of this page. For example, using the Issues tab, users can report bugs or leave comments.

Among the family of software packages for configurational data analysis, **CORA** is the only package almost completely written in Python. It can thus be used in a purely code-based Python environment. Alternatively, **CORA** can be run in a Google Colaboratory notebook, or simply ”Colab notebook”, which provides a graphical environment. Since we expect that the majority of **CORA** ’s potential users will prefer a graphical interface, we will focus on this type of environment in the remainder of our tutorial.

A Colab notebook is a web-integrated development environment that can combine executable code, rich text, pictures, HTML, and LaTeX, among many other components.^6^ As Colab notebooks run on Google cloud servers, the power of Google’s hardware, including graphics and tensor processing units, can be utilized. Additional computing power can be bought at different plans. The infrastructure of Colab notebooks is therefore particularly geared towards collaborative projects on data science.

**Figure 2.**
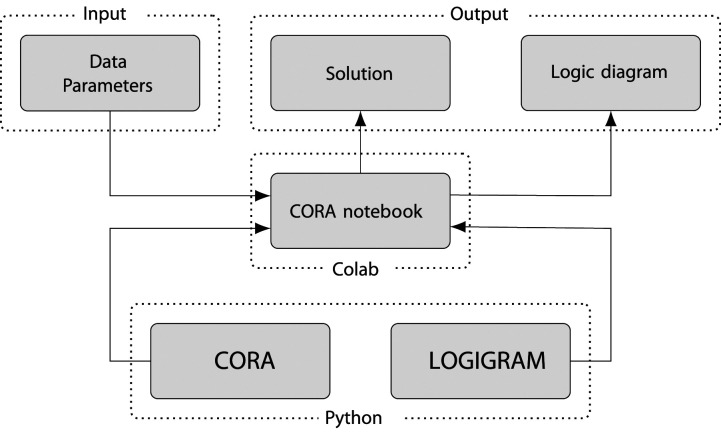
The internal structure of **CORA** (Sebechlebská et al., 2023, p. 2).

Figure 2 shows the internal structure of **CORA** . At the lowest level, two distinct Python packages constitute its foundation. While **CORA** is dedicated to data processing and optimization algorithms developed under CORA’s methodology, **LOGIGRAM** implements CORA’s graphical component—the production of logigrams. On the middle layer, these two Python packages are combined in CORA’s Colab notebook, which provides the visual interface to the user. This interface represents the top level of **CORA** on which all interactions between the user and software take place. The input the user has to provide consists, first and foremost, of a set of CORA-compatible data. In addition to data, **CORA** requires the specification of certain modelling and optimization parameters.

In line with CORA’s reliance on two distinct Python packages, two types of output can be generated: a textual solution, which consists of a (system of) Boolean function(s) expressed in the syntax of propositional logic, and a logigram of a (system of) Boolean function(s).

**Figure 3.**
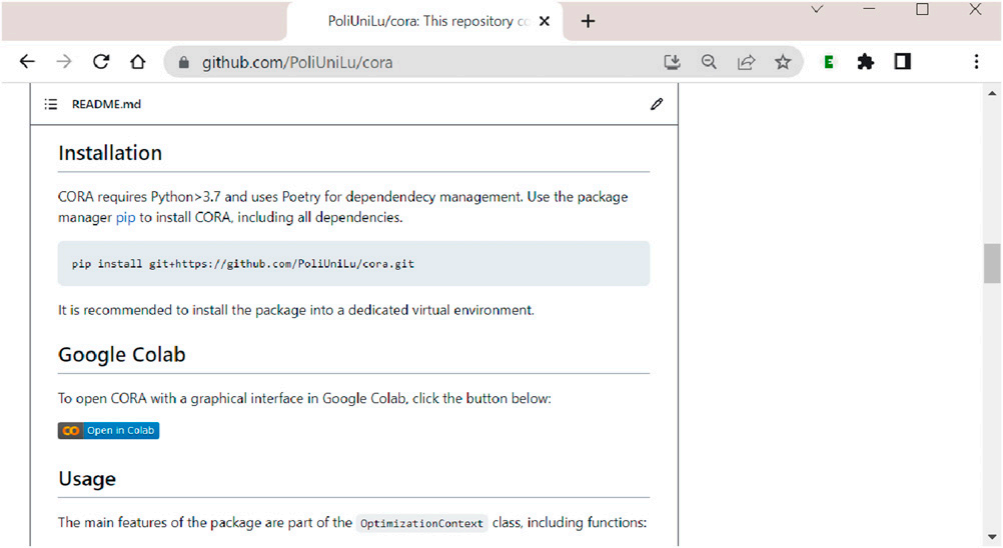
Launching **CORA** ’s Colab notebook from its GitHub page.

From **CORA** ’s page on GitHub, users can go directly to **CORA** ’s Colab notebook, either by clicking the ”Open in Colab” badge in the badge section above CORA’s logo (see Figure 1) or by clicking the ”Open in Colab” badge under the section ”Google Colab” as shown in Figure 3. A Google account is required to use the notebook, which is the only limitation, but once registered, CORA can be used on any operating system as well as in any modern web browser.

**Figure 4.**
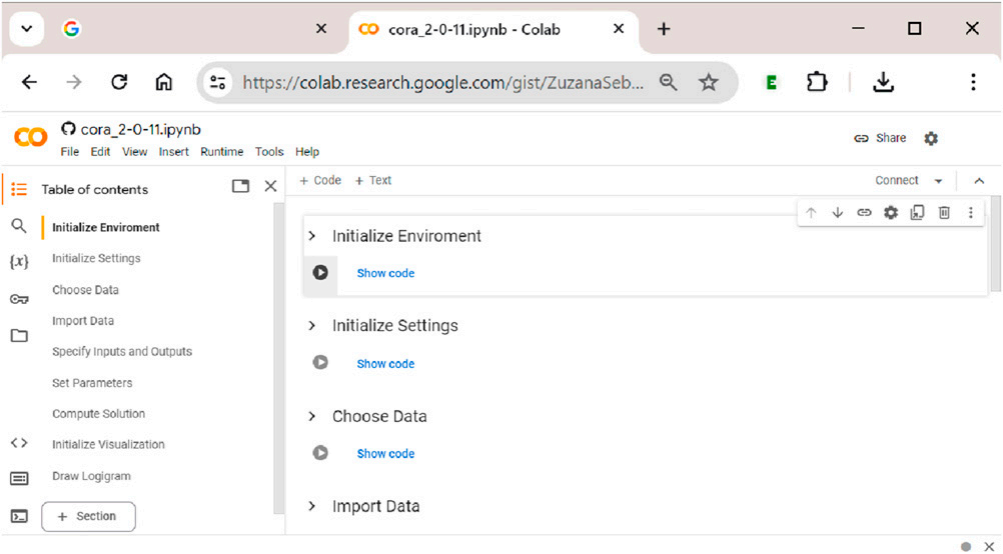
Procedural sections in **CORA** ’s Colab notebook.

After having clicked the ”Open in Colab” button, the user is taken to **CORA** ’s Colab notebook, which is shown in Figure 4. The workflow is meant to guide users through the analysis, which comprises nine sections: (1) the initialization of the environment and (2) default settings, the (3) choice and (4) import of data, the (5) specification of the inputs and outputs, (6) the setting of search parameters and thresholds for data fit, (7) the computation of the solution, (8) the initialization of **CORA** ’s visualization module and, finally, (9) the drawing and export of logigrams.

Table 2 lists all these sections together with a brief description of their purpose. Sections 1–2 set up the notebook environment and settings, sections 3–6 cover data-related preparations and modelling parameters. In section 7, the optimization procedure is run, and all solution-related output is generated. Sections 8–9 cover all functionality for the visualization of solutions.

**Table 2.** Procedural Sections in **CORA** and Their Purpose.

#	Section	Purpose
1	Initialize Environment	Load **CORA** package into Colab environment
2	Initialize Settings	Generate notebook defaults and settings
3	Choose Data	Choose dataset to be imported
4	Import Data	Import data as csv or xls(x) file
5	Specify Inputs and Outputs	Determine case labels, inputs and outputs
6	Set Parameters	Set all parameters for optimization
7	Compute Solution	Produce solution and fit statistics
8	Initialize Visualization	Load **LOGIGRAM** package into Colab environment
9	Draw Logigram	Generate logigram of solution

## How to Use **CORA** ’s Colab Notebook: an Empirical Example

**Figure 5.**
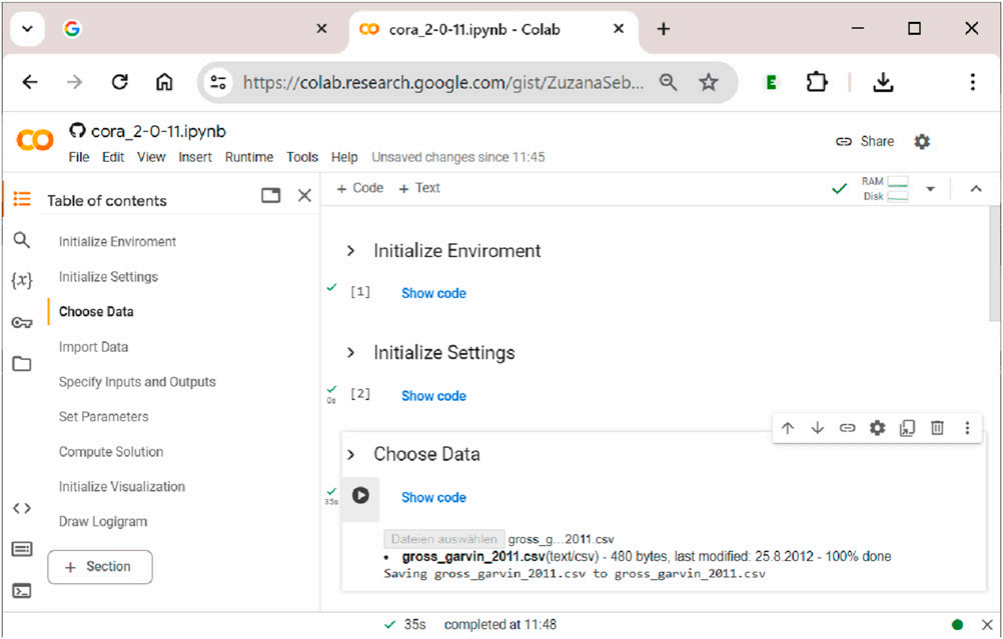
Initializing **CORA** ’s sections and choosing data.

In the following, we demonstrate in a hands-on empirical example how to use **CORA** ’s Colab notebook and how to interpret its outputs. To this end, we sub-structure this article section along the procedural sections of the notebook. Every section of **CORA** ’s notebook needs to be initialized when running the notebook for the first time within a session. This is done by clicking the right-pointing ”Run” button below the heading of each section cell (see Figure 4 again). Once this initialization process has succeeded, a green tick mark will be displayed. This is shown in Figure 5.

Every time a new session is started, sections 1 (Initialize Environment) and 2 (Initialize Settings) need to be re-run, but within the same session, this is not necessary. If the user wants to only change the data, the inputs or outputs, the analytical parameters or any other component along the way from section 3 (Choose Data) to section 7 (Compute Solution), a re-initialization from the first concerned section is required before the process can be restarted and the section’s output be updated.

Only sections 8 (Initialize Visualization) and 9 (Draw Logigram) can be used without initializing any previous sections because they import and provide the functionality of the **LOGIGRAM** package as an independent module. In that way, users who have generated a configurational solution with other software, such as a QCA solution with **QCApro** or a CNA common-cause structure with **cna** in **R** , for instance, can directly produce a logic diagram of their solution without having to re-run the analysis in **CORA** .

### Sections 1–4: Initialization and Data Upload

**Figure 6.**
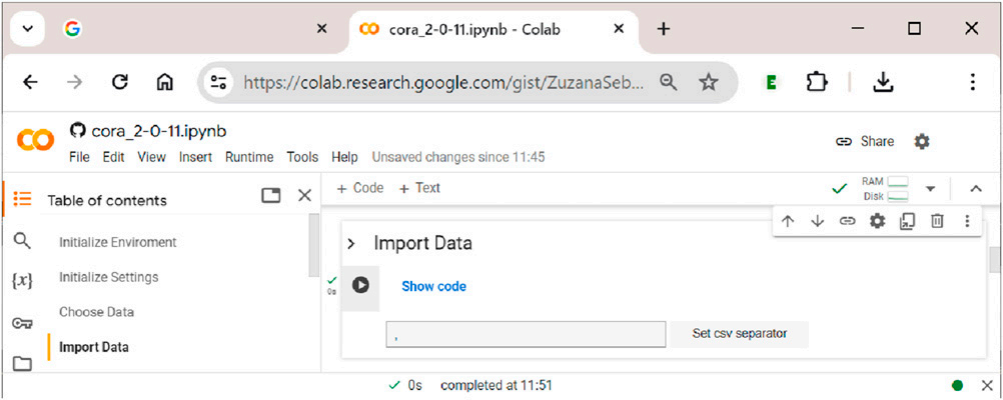
Importing data into **CORA** ’s notebook.

Every time a new session is started, sections 1 ”Initialize Environment” and 2 ”Initialize Settings” need to be run first. This ensures that the **CORA** package is loaded into the Colab environment and that the notebook’s default values and settings apply. The next step is to upload the data into **CORA** . To this end, the user first has to choose a dataset in section 3 ”Choose Data” (in csv or xls(x) format), and then import it in section 4 ”Import Data”. The first of these two steps in uploading data to **CORA** is shown in Figure 6, where the dataset ”gross_garvin_2011.csv” is the dataset from the study by Gross and Garvin (2011) on the success of public-private partnerships in toll-road contracts in achieving their public pricing objectives.^7^ We have chosen this dataset simply because it is relatively small and thus typical of many datasets in configurational research, it includes multi-value inputs, and it features three distinct outputs.

**Figure 7.**
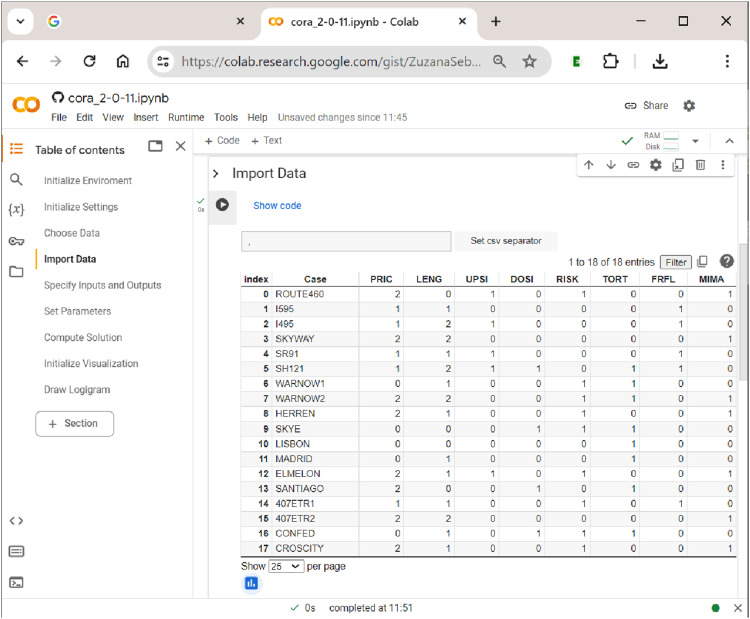
Data display in **CORA** ’s notebook after import.

The second step in uploading the data is shown in Figure 7. Here, the separator of the csv file needs to be specified, which is usually a comma (but can sometimes also be a semicolon). By clicking the button ”Set csv separator”, the separator is determined, and the file is properly read into the notebook (if the wrong separator is used, the data will not display as a properly aligned table).

Gross and Garvin (2011) study the effectiveness of public–private partnership contracts for toll roads. To do so, the authors employ multi-value QCA. Their five exogenous factors include the toll rate approach (PRIC; 0 = average cost pricing, 1 = marginal social-cost pricing, and 2 = revenue-maximizing pricing), the concession length (LENG; 0 = variable, 1 = short, and 2 = long), upside revenue sharing (UPSI; 0 = absent and 1 = present), downside risk sharing (DOSI; 0 = absent and 1 = present), and the traffic-demand risk (RISK; 0 = low and 1 = high). Two endogenous factors are related to pricing objectives: achieving an affordable/specific toll rate (TORT: 0 = low, 1 = high) and managing congestion or maximizing throughput (FRFL: 0 = low, 1 = high). Originally, the dataset used by Gross and Garvin (2011) contains three endogenous factors, each of which is analyzed separately with mvQCA, but we use only two here for the sake of keeping the complexity of the simultaneous output analysis with CORA at a purposeful level.

### Section 5: Specifying Inputs and Outputs

**Figure 8.**
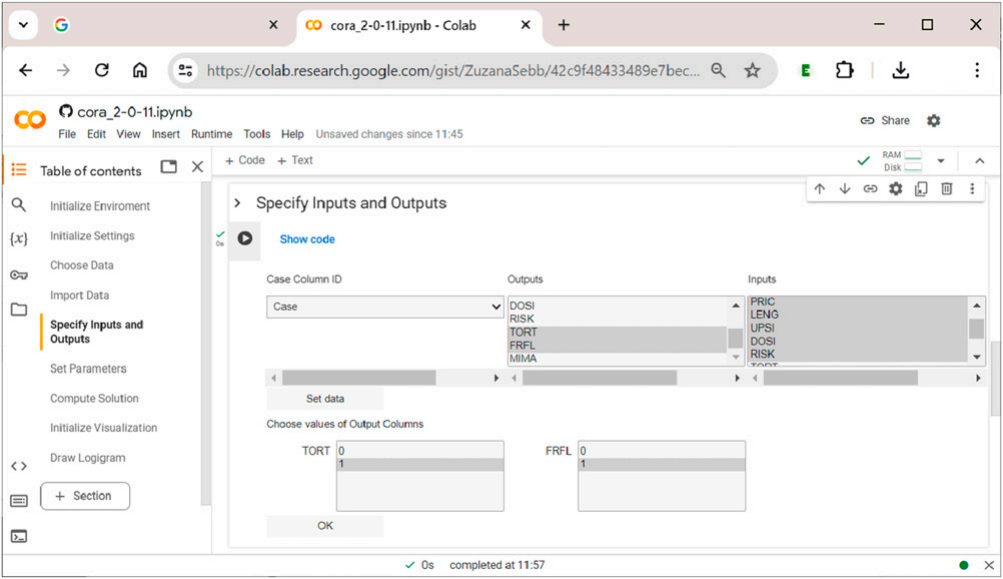
Specifying inputs and outputs in **CORA** ’s notebook.

After having imported the raw data, the fifth section of **CORA** ’s notebook asks the user to specify the inputs and outputs. At least one input and one output must be selected from the factors available in the data.^8^ Simultaneously, the user can also provide the name of the column in the data that contains the case labels. Once the user has confirmed the choices, the respective outcomes that should be analyzed must be selected. This is shown in Figure 8, where the value ”1” of output TORT and the value ”1” of output ”FRFL” has been selected. As all three outputs in Gross and Garvin’s data are binary, either the value ”0” or ”1” has to be chosen for the outputs selected previously. However, had any output been a multi-value factor, the user could have chosen a single value (e.g., ”0”, ”1”, ”2”,…), or even a combination of values (e.g., ”0” and ”1”).

After all required specifications have been provided, a table that shows the re-coded raw data according to the user’s settings is displayed for brief visual cross-checking (not displayed here).

### Section 6: Setting the Parameters of the Analysis

**Figure 9.**
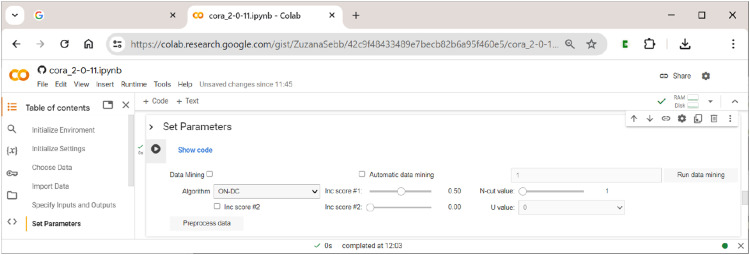
Setting the parameters of the analysis.

In this section, the user sets all parameters of the analysis, chooses the algorithm that will perform the optimization, and pre-processes the data to generate (multi-output) truth tables on which the chosen algorithm will operate. In addition, this section includes the option to use the data-mining feature of CORA. How these options are arranged in **CORA** is shown in Figure 9.

The main analytical parameters for the generation of truth tables largely correspond to those also used in QCA and CNA. They are as follows:

• Inc score #1: This score corresponds to what is often called the ”consistency cut-off” in QCA and CNA, and separates positive from negative terms in the truth table (if a term is positive, it means that this term is sufficient for the respective outcome); it should not be below 0.5. Note how this is different from standard practice in QCA, where consistency cut-offs of 0.8 or even higher are often required, which may lead to considerable problems of overfitting (Arel-Bundock, 2022; Baumgartner, 2022).
• Inc score #2: This score is a numeric value between 0 and the value of Inc score #1. Terms falling into this range are assigned the value U. In this way, users can quickly test the effect of changing the function value of a term from 1 to 0 or vice versa for certain ranges of inclusion. For example, for inclusion scores between 0.5 (Inc score #2) and 0.6 (Inc score #1), U = 1 has the same effect as setting Inc score #1 to 0.5 without using Inc score #2, whereas U = 0 has the same effect as setting Inc score #1 to 0.6 without using Inc score #2. This simply provides a quicker way to run two separate analyses.
• N-cut value: This value is a case frequency cut-off that determines the minimum number of cases below which the term is considered unobserved.
• Pre-process data: Pressing this button will generate and print the truth table. Note that it must not be pressed when the intention is to use data-mining, in which case the button ”Run data mining” should be used.

In the current version of **CORA** (2-0-11), two optimization algorithms are implemented: ON-DC and ON-OFF. ON-DC is based on the multi-outcome version of the original Quine-McCluskey algorithm (QMC) (McCluskey, 1965). To generate the solution, this algorithm operates on all positive terms and all terms which are not defined in the truth table - the so-called don’t care (DC) terms (for a worked example, see Thiem et al., 2024). The simple single-outcome version of QMC is also the core algorithm of QCA (Ragin & Davey, 2019). ON-OFF, in contrast, is based on the multi-outcome version of McCluskey’s alternative to QMC (McCluskey, 1962). It operates only on the positive (ON) and negative (OFF) terms of the truth table, and does not require DC terms (for a worked example, see Mkrtchyan et al., 2023).

Note that, despite their different approach, both algorithms are completely equivalent and thus produce exactly the same solutions. The fact that both are available in **CORA** nonetheless has a methodological and a technical aspect. Methodologically, it demonstrates that the entire body of literature on logical remainders (DC-terms) in QCA has been a mis-selling of QMC from the start. Had QCA’s inventor Charles Ragin not chosen QMC, but McCluskey’s own alternative ON-OFF algorithm, no debate on logical remainders and their associated procedures, such as conservative or intermediate solutions in QCA, would have ever come up. Thus, and contrary to QCA, DC-terms must never be manipulated in CORA because such manipulations would lead to high rates of false inferences (cf. Baumgartner & Thiem, 2020; Thiem, 2022a). Technically, and more importantly, the ON-OFF algorithm enjoys a significant computational advantage when the size of the DC-set is large relative to the size of the OFF-set, whereas the ON-DC algorithm enjoys significant computational advantages when the size of the OFF-set is large relative to the size of the DC-set, given a fixed size of the ON-set. If an analysis in **CORA** proves difficult to solve with one of the algorithms, users may thus want to try the respective algorithmic alternative.

Configurational data-mining is one of the main distinctive features of CORA. The basic idea behind this approach is that any solution that is found with a given number of inputs, must, *ceteris paribus*, also always be found in an analysis with only those inputs present in the system. This approach to selecting inputs has first been tested in the context of QCA (Haesebrouck & Thiem, 2018; Lankoski & Thiem, 2020), but CORA is the first CCM to offer an in-built and systematic procedure to analyze all n-tuples of input combinations in a search for feasible tuples of solution-generating inputs. In essence, this feature thus provides a configurational version of Occam’s Razor, which says that explanations that involve fewer variables are, *ceteris paribus*, to be preferred over explanations that are more complex (Feldman, 2016). For more information on further practical considerations behind this approach, we refer readers to the original presentation of CORA’s methodology in Thiem et al. (2022).

**Figure 10.**
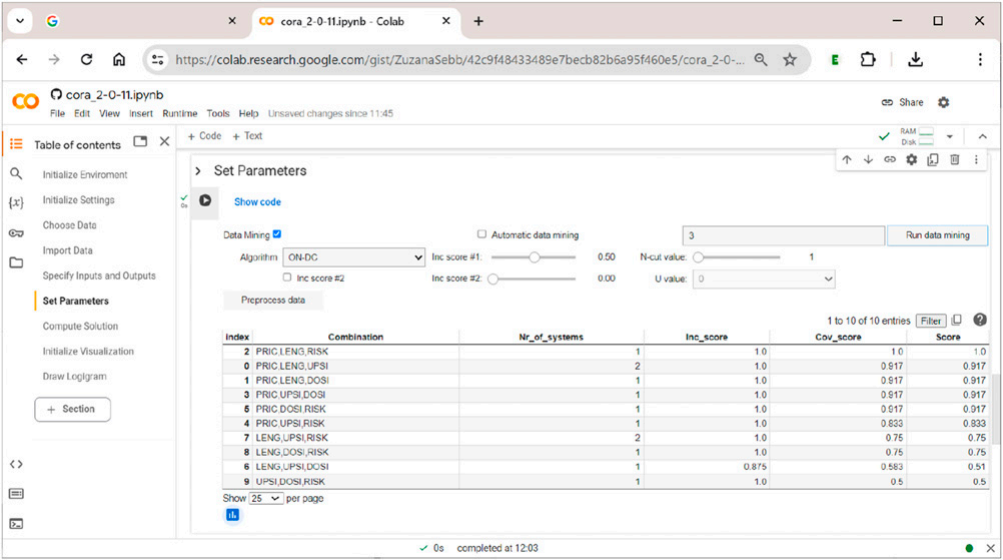
The data-mining feature of **CORA** .

To run data-mining, the user needs to tick the respective checkbox ”Data Mining”, to provide the number of exogenous factors and to indicate the preferred optimization algorithm in the corresponding drop-down menu. Alternatively, by ticking ”Automatic data mining”, the user can let the software search for the minimum size of a factor combination that passes the specified cut-offs (the standard starting value is 1 input). For example, when running (non-automatic) data-mining with tuples of three exogenous factors, **CORA** will return the table shown in Figure 10.

There are ten possible ways to form tuples of three exogenous factors from a set of five exogenous factors. Thus, the table has ten rows (row index counting starts with 0). The respective combinations of factors are given in the column ”Combination”. The column ”Nr_of_systems” shows the number of systems that result under the respective choice of factors. In the column ”Inc_score”, the solution’s inclusion score is given. Its coverage score is shown in the column ”Cov_score”. And finally, the product of the solution’s inclusion and coverage score is listed in the column ”Score”.

In this example, there is one combination of exogenous factors that clearly performs best: the combination of factors PRIC, LENG, and RISK (index #2). Note that this information does not yet provide any information on how these factors and their values are related to each other inside the solution. It just says that, when choosing these factors as inputs, the solution will provide the best fit to the data. In addition, this combination of factors yields only one system, whereas other combinations also yield a single system, yet score lower on data fit, while still others (e.g., PRIC, LENG, and UPSI; index #0) yield two systems and have lower data fit at the same time. Generally speaking, there is an inherent trade-off between these indicators. The larger the size of the combination, the higher the data fit tends to become, but the higher will also be the number of alternative systems fitting the data equally well. It thus makes little practical sense to try and increase data fit by small margins when the price is a significant rise in ambiguity.

After the output table of **CORA** ’s data-mining procedure has been evaluated, the preferred combination can then be chosen in the list of inputs in the section ”Specify Inputs and Outputs”.^9^ Subsequently, the user proceeds to the ”Set Parameters” section again and clicks the button ”Preprocess data” this time to generate the (multi-output) truth table.

**Figure 11.**
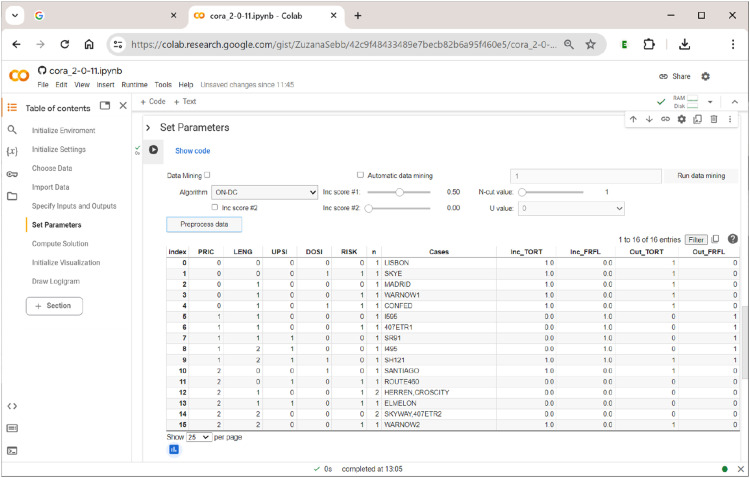
A multi-output truth table in **CORA** .

For demonstration purposes, however, we continue with all five exogenous factors that have also been part of the analyses of Gross and Garvin (2011) and that we have provided in the section ”Specify Inputs and Outputs” (thus, if data mining is used with the same number of inputs that have been initially provided in the section ”Specify Inputs and Outputs”, there will only be one combination of factors). The corresponding truth table for both outputs TORT and FRFL is shown in Figure 11.

What is unique in CORA with respect to truth tables is that a separate function value is assigned to each output (Out_TORT, Out_FRFL) based on each term’s respective inclusion value (Inc_TORT, Inc_FRFL). With two outputs, there thus exist four different effect constellations: 00 (no outcome present), 10 (only first outcome present), 01 (only second outcome present) and 11 (both outcomes present). Had we also added the authors’ third endogenous factor (MIMA), there would have been eight different effect constellations (000, 001, 010, 100, 110, 101, 011, 111). The complexity of the analysis, and also the demands on the user’s ability to interpret the findings, therefore doubles with each output added. In the case of Gross and Garvin’s data, there is only a single case (Index #9; case SH121) where both outcomes are present. In all other cases, only one is present or none is present.

### Section 7: Computing and Interpreting Solutions

**Figure 12.**
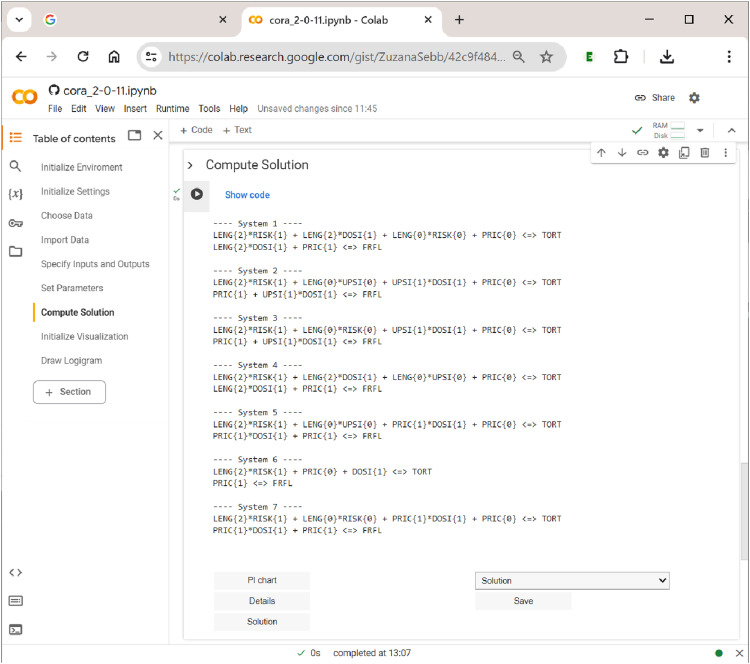
Computing solutions in **CORA** .

In CORA, the solution consists of the set of all irredundant systems (cf. Thiem et al., 2022, p. 8). It is computed in the section ”Compute Solution”. As soon as the ”Run” button of this section is being operated, **CORA** applies the algorithm chosen in the previous section on the (multi-output) truth table and generates the solution in the background. Note again that using the ON-DC algorithm will result in exactly the same solution as the ON-OFF algorithm. Only the speed whereby solutions are generated will differ. To see the solution in plain textual form, the button ”Solution” can be used. This is shown in Figure 12.

In the case of the analyzed data by Gross and Garvin (2011), the solution consists of seven systems. Analogously to the interpretation of simple models for single outcomes, these systems represent alternative explanations of the same data that fit those data equally well. Sometimes, these systems may be relatively similar to each other, but sometimes, they may also differ fundamentally. A straightforward way to check the degree to which systems are different from each other is to navigate to the dropdown menu above the ”Save” button and choose ”Solution structure”. This will download a Microsoft Excel spreadsheet in which the prime implicants (PIs) are listed across the columns and the outcomes and systems across the rows. A ”1” entry indicates that the PI is a PI for a certain outcome in a certain system. With such an occurrence matrix, users can easily implement their own diagnoses with Excel’s internal filters and functions.

A system returned as part of a solution in CORA must be interpreted in the following way: All PIs that are unique to an outcome represent parts of causal paths to that outcome only, while all PIs that are shared between outcomes represent parts of causal paths to the conjunction of these outcomes only. For instance, in system 1, LENG{2}DOSI{1} is a PI that is shared by TORT and FRFL. That means the former is part of a shared cause of a complex effect that involves both TORT and FRFL. In contrast, for example, PRIC{0} is unique to TORT, while PRIC{1} is unique to FRFL. These PIs thus represent parts of causal paths that lead to the respective outcome in the absence of the other outcome.

However, it is possible that, although the data contain cases showing a conjunction of two or more outcomes, these outcomes need not necessarily form a complex effect with a shared cause. For instance, although case SH121 in Gross and Garvin’s data shows both TORT and FRFL, system 6 provides an explanation in which these two outcomes have nothing to do with each other. Each effect is individually explained by distinct causes. It is one of the major advantages of CORA that the method is able to reveal whether a conjunction of two or more effects is systematically linked to shared causes, or whether this conjunction may merely be a coincidence of two unrelated effects.

**Figure 13.**
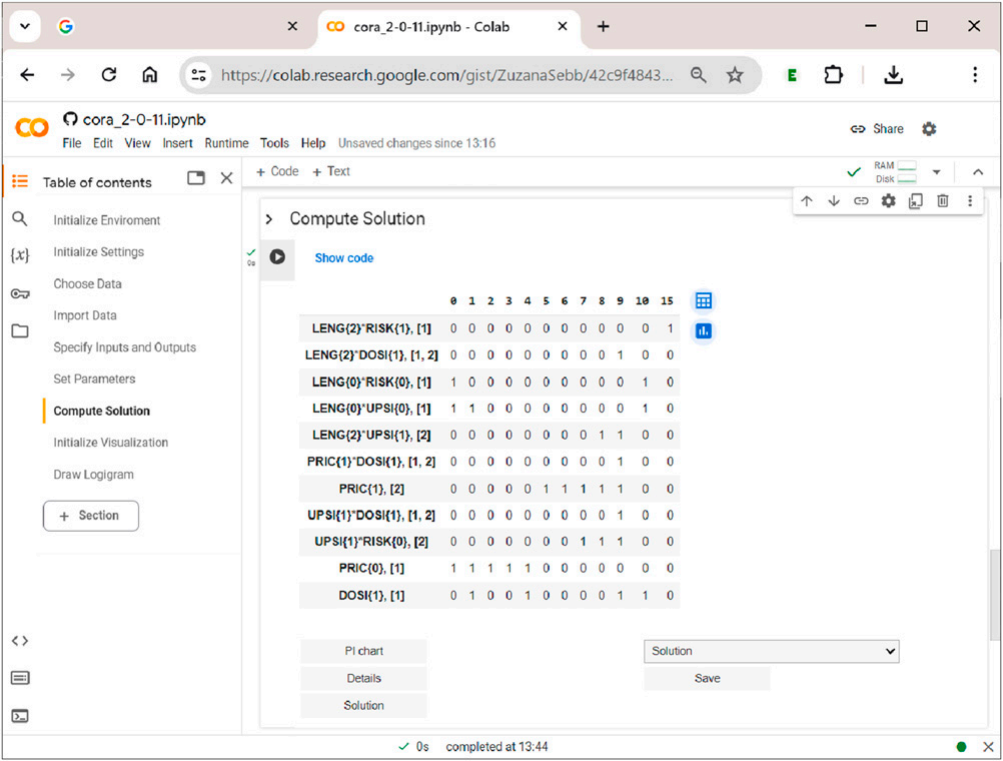
The prime implicant chart in **CORA** .

Last, but not least, the user has the option to display the PI chart, which is shown in Figure 13. In square brackets, each PI has an index attached which provides the outcomes to which that PI refers. PIs with more than one index number are proper multi-output PIs and thus potential candidates for a shared cause of a complex effect. For example, in Figure 13, three PIs—LENG{2}DOSI{1}, PRIC{1}DOSI{1}, and UPSI{1}DOSI{1}—-are shared between the two outcomes and therefore have the index ” $\left[1,2\right]$ ” attached. In the dropdown menu above the ”Save” button, users can also directly download the PI chart as an Excel spreadsheet, either for further analysis or inclusion as a supplementary file in a publication.

**Figure 14.**
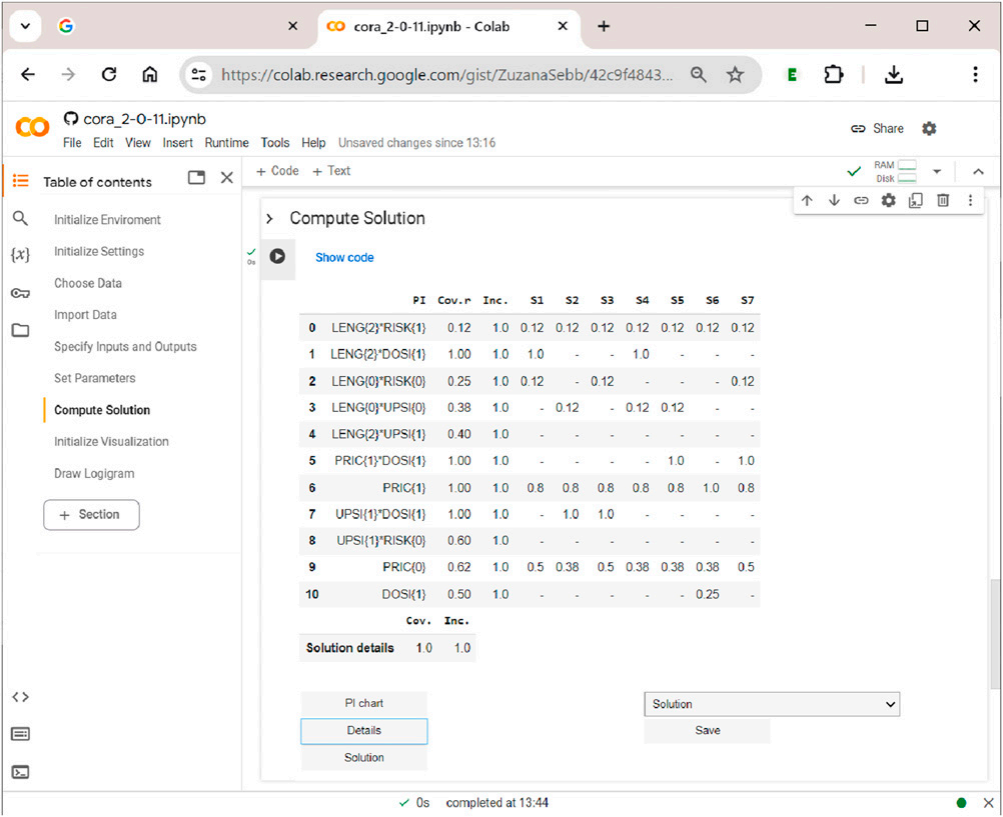
The solution details in **CORA** .

Of interest to the user are also the details of the solution, mainly inclusion and coverage values, both raw and unique. The table of details, which is shown in Figure 14, lists all PIs that have been part of at least one system across the rows, and the different systems across the columns. The first column after the PI column gives the PI’s raw coverage, the second column its inclusion. Below each system, unique coverage values are provided. For PIs that are unique to an outcome, these values are calculated as usual. However, for PIs that have more than one output index, values are calculated with respect to all constellations to which the PI refers. For example, under systems 1 and 4, LENG{2}DOSI{1} is the only shared PI of the complex effect TORT{1}FRFL{1}. For that reason, its unique coverage is 1.

### Sections 8–9: Drawing Logigrams

The last part of **CORA** ’s notebook deals with the visualization of results. To this end, **CORA** draws on logic diagrams, which are more succinctly called ”logigrams” in CORA because they use a small and standardized set of graphical components (Thiem et al., 2022). As we have already argued elsewhere why logic diagrams are useful in the context of Boolean causal inference and configurational data analysis (Thiem et al., 2023), we focus on showing in this article how users can create professionally looking logigrams in **CORA** .

Since the **LOGIGRAM** package is a stand-alone Python module, the visualization section requires a separate initialization. Once this has been executed, the next section ”Draw logigram” first asks whether the expression is to be specified manually (Input System) or whether a system from the previous analysis should be used (Choose System). If an analysis has been carried out before, it is easier to simply choose a system instead of a manual input. The option to input systems manually is primarily meant for users who want to enter a suitable Boolean or multi-value expression from an external analysis.

**Figure 15.**
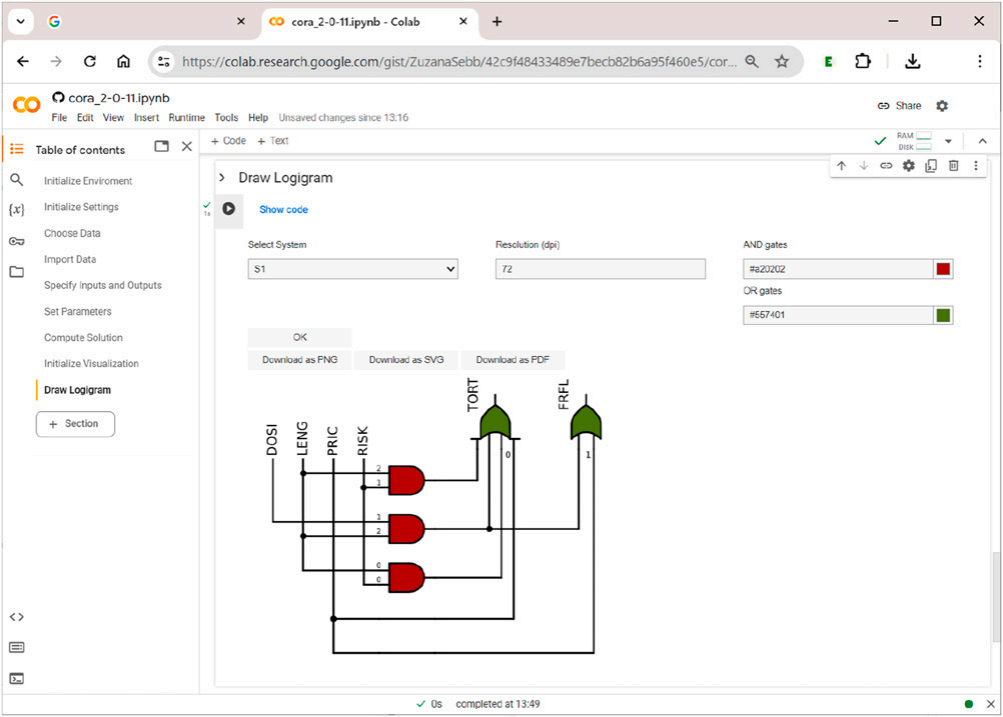
Drawing logigrams in **CORA** .

Once the user has opted for the choice of a system from the solution and has picked the desired system, pressing the ”OK” button will generate a plain black-and-white logigram. This can be enhanced with colours for gates. Finally, users can download the respective figure as a png picture file with a specified resolution (default 72 dpi), or as a vector file in svg or pdf format. Figure 15 shows the logigram of the first system (S1) among the seven systems that have resulted from the analysis of Gross and Garvin’s data (cf. Figure 12).

Note that only one system can be rendered as a logigram at a time. If the system to be visualized does not contain conditions or outcomes from multivalent factors, users can choose between case-based notation (e.g., *A* and *a*) and prime-based notation (e.g., *a* and *a*′).

## Conclusions

Modern Configurational Comparative Methods have gained in popularity among social scientists over the last thirty years. Combinational Regularity Analysis (CORA) has recently joined this family of methods. In this article, we have provided a software tutorial for the open-source package **CORA** , which implements the eponymous method. In particular, we have demonstrated how to use **CORA** to discover shared causes of complex effects and how to interpret corresponding solutions correctly, how to mine configurational data to identify minimum-size tuples of solution-generating inputs, and how to visualize solutions by means of logigrams. With all this functionality, **CORA** represents a significant addition to the landscape of software packages for conducting configurational data analysis.

Future versions of **CORA** will feature further enhancements. For example, the current version (2-0-11) offers only two exact optimization algorithms, that is, algorithms whose search target represents a global optimum. However, aiming for a global optimum limits the size of the problems that can be solved. Given the current drive towards ”big data” in many disciplines of the social sciences, at least one approximate optimization algorithm will thus be added in a future version. In addition, the production of logigrams will be facilitated and further improved.

## Notes

1. Note that the principle of Occam’s Razor does not imply that more complex explanations never reflect the truth.

2. With respect to QCA, for example, the authors of reviews of and comparisons between QCA software were not able to find out why some packages such as **fs/QCA** or **Tosmana** produced solutions different from those of **QCApro** or **QCA** despite identical data and parameter settings (Baumgartner & Thiem, 2017; Thiem & Duşa, 2013a). Other examples that underline the need for open source are not difficult to find. For instance, it has recently been shown in medical research that the most popular software for spatial extent analysis in functional magnetic resonance imaging (fMRI) produced unacceptably high false-positive rates (Eklund et al., 2016); and in the social science, the editors of the journal *European Union Politics* learned only after a long search process that the company of the statistical software that was used by an author of an accepted manuscript had changed an algorithm from one software version to the next, as a result of which all attempts of replicating the statistical analysis of the manuscript had failed (Ershova & Schneider, 2018).

3. Particularly in QCA, the use of multivalent factors, called multi-value QCA (mvQCA), is not common. Some works have speculated about the reasons for the niche existence of mvQCA (Thiem, 2013, 2015).

4. Note how this approach of CORA compares with that of QCA. In QCA, whose optimization paradigm rests on the Quine-McCluskey algorithm, applied researchers have been advised to deviate from QMC’s algorithmic protocol and to manipulate don’t care terms prior to Boolean optimization. Such practices are neither allowed in CNA nor in CORA since these manipulations are tantamount to adding artificial data, which in turn leads to a demonstrable inflation of false positive inferences (Baumgartner & Thiem, 2020; Thiem, 2022a; Thiem & Mkrtchyan, 2024b).

5.
https://github.com/PoliUniLu/LOGIGRAM

6. More precisely, Colab notebooks are Jupyter-Notebooks hosted by Google.

7. Users wishing to re-run the demonstration with these data can download them from the article’s website as supplementary material.

8. Note that there is no methodological limit in CORA on the number of inputs, as is the case in QCA (Thiem & Mkrtchyan, 2024a). The more inputs that are added, however, the higher the level of ambiguity tends to become (cf. Baumgartner & Thiem, 2017).

9. In future versions of **CORA** , we seek to streamline this process by linking the two sections.
